# Age-Group-Specific Associations between the Severity of Obstructive Sleep Apnea and Relevant Risk Factors in Male and Female Patients

**DOI:** 10.1371/journal.pone.0107380

**Published:** 2014-09-11

**Authors:** Xingqi Deng, Wei Gu, Yanyan Li, Mei Liu, Yan Li, Xiwen Gao

**Affiliations:** 1 Department of Emergency Medicine, the Center Hospital of Minhang District, Minhang District, Shanghai, China; 2 Department of Respiratory Medicine, the Center Hospital of Minhang District, Minhang District, Shanghai, China; National Institute for Viral Disease Control and Prevention, CDC, China

## Abstract

**Aim:**

To seek accurate and credible correlation manner between gender, age, and obesity; and the severity of obstructive sleep apnea (OSA) in large-scale population.

**Methods:**

Totals of 1,975 male and 378 female OSA patients were sequentially recruited. Centralized covariant tendencies between age, body mass index (BMI), and waist hip ratio (WHR); and OSA severity, were explored in a gender-specific manner via multiple statistical analyses. The accuracies of observed correlations were further evaluated by adaptive multiple linear regression.

**Results:**

All of age, BMI, WHR, smoking, drinking, and OSA severity differed between males and females. BMI and WHR were positively and (approximately) linearly associated with OSA severity in both males and females. Restricted cubic spline analysis was more effective than was the Pearson correlation approach in correlating age with AHI, and provided age crossover points allowing further piecewise linear modeling for both males and females. Multiple linear regression showed that increasing age was associated with OSA exacerbation in males aged ≤40 years and in females aged 45–53 years. BMI, WHR, and diabetes were independently associated with OSA severity in males with age-group-specific pattern. In females, only BMI was associated with OSA severity at all ages.

**Conclusions:**

In male patients, BMI and WHR are prominent risk factors for OSA exacerbation. Age and diabetes are associated with OSA severity in males of particular ages. In females, BMI is also a prominent risk factor for severe OSA, and OSA severity increased with age in the range 45–53 years.

## Introduction

OSA is increasingly recognized as an intricate manifestation of several systemic disorders, creating a disease that directly threatens the basic life processes of breathing and sleep [1]. OSA is characterized by repeated episodes of upper airway obstruction during sleep, causing expiratory dyspnea, intermittent oxygen desaturation, systemic and pulmonary arterial blood pressure surges, and sleep disruption [2]. Long-term chronic intermittent hypoxia, hypoxemia, and sleep fragmentation causes and aggravates cardiovascular and cerebrovascular diseases [1], [3], [4], metabolic syndrome [5], diabetes [6], resistant hypertension [7], and cognitive disorders [8]; and reduces the quality-of-life [9]. Therefore, OSA exacerbates several pathophysiological processes and constitutes a major disease burden worldwide.

Factors recognized to increase the risk of OSA development include aspects of upper airway anatomy and function (e.g., nasal obstruction and large tonsils) [10]; ethnic origin (African American, Asian, and Native American) [11]; endocrine components (e.g., an underactive thyroid gland, menopause, and higher levels of testosterone); and personal habits (e.g., the use of alcohol, tobacco, and sedatives) [11], [12]. Other well-known risk factors include male gender [13], age [14], and obesity [15] were associated with OSA in observational studies performed on either general populations or OSA patients. In a 283-participant family case-control study, the AHI was significantly associated with age (OR per 10-year increase in age, 1.79), BMI (OR per 1.0 unit of increase, 1.14), WHR (OR per 0.1 unit of increase, 1.61), and gender (OR for males vs. females, 4.12), upon ordinal logistic regression analysis [16]. Risk factors for OSA in 450 males and females with congestive heart failure varied by gender; in males, only BMI was significantly associated with OSA (OR for BMI>35 kg/m^2^, 6.10), whereas, in females, age was the only important risk factor (OR for age>60 years, 6.04) [17]. A study using a two-phase random sample from a general population showed that the ratio of sleep apnea in males compared to females was 3.3∶1; the prevalence of sleep apnea was rather low in premenopausal and postmenopausal females undergoing hormone replacement therapy (HRT); the prevalence of sleep apnea in postmenopausal females not taking HRT approached that of males; and, in premenopausal females, the presence of sleep apnea appeared to be associated exclusively with obesity (BMI>32.3 kg/m^2^) [13]. Another two-stage study performed on a general male population showed that the prevalence of sleep apnea tended to increase with age, but severity decreased [14]. More detailed studies showed that the increased prevalence of OSA in the elderly appeared to plateau after the age of 65 years [18]. However, when the data were controlled for BMI, severity appeared to decrease with age [18].

In summary, many studies have shown that age, gender, and obesity are associated with OSA, and, although the precise contributions made by these factors to OSA risk remain to be determined, other issues also require attention. First, many studies insufficiently correlated the variables mentioned above with OSA prevalence either in small or specific populations, and the conclusions drawn may thus be in error. Second, both community and patient populations had limited ranges of OSA severity (AHI ≤30), and most studies did not consider the extent of OSA severity encountered in clinical practice. Many clinical patients have severe conditions, with AHI values >30. Third, many studies simply categorized subjects into mild, moderate, and severe OSA groups, and did not plot variables against real OSA severity index values, such as the AHI, thus losing relevant information.

To seek accurate and credible correlation manner between gender, age, and obesity; and the severity of obstructive sleep apnea (OSA) in large-scale population, in the present study, we recruited a total of 2,353 OSA patients (1,975 males and 378 females) with AHI values ranging from 5 to 100. Centralized covariant tendencies between age, gender, and obesity; and OSA severity were profiled by scatter diagrams, data conversion, Pearson correlation, stratification and restricted cubic spline analyses. Age-group-specific associations between the severity of OSA and relevant risk factors in male and female patients were revealed by adaptive multiple linear regression.

## Materials and Methods

### Patients

This study was conducted in accordance with the amended Declaration of Helsinki. The Review Board of the Center Hospital of Minhang District approved the study protocol (reference number: SHMHCH 2008–0012). Written informed consent was obtained from all patients according to the guidelines of the Chinese National Ethics Regulation Committee; we explained the procedure to all patients and emphasized that their data would be used in this study. All patients were informed of their rights to withdraw consent either personally; or via kin, caretakers, or guardians.

An OSA patient referred for diagnosis was first screened by a single physician and next subjected to whole-of-night polysomnography. We sequentially recruited 2,749 OSA patients treated in our hospital from 2008 to 2013. Exclusion criteria were: a sleep disorder other than OSA; subjects have been treating with continuous positive airway pressure; unstable cardiopulmonary disease; chronic kidney disease; and hormone treatment. Finally, 2,353 adult patients (1,975 males and 378 females) aged 20–80 years were included in this study.

### Data collection

Polysomnographic (Somnostar 4100, Sensormedics) data included those derived via electroencephalography; electrooculography; submental electromyography; electrocardiography; nasal and oral airflow measurements; assessment of chest and abdominal respiratory movements; and oxyhemoglobin saturation levels. An abnormal breathing event was defined as complete cessation of airflow (apnea) for more than 10 s or a >50% reduction in respiratory airflow accompanied by a decrease of ≥4% in oxyhemoglobin saturation (SaO_2_), and/or an electroencephalographic arousal. The diagnosis of OSA was based on patient complaints, medical history, and integrated data (apnea-hypopnea, oxygen desaturation, and arousal) yielded by overnight polysomnography [19]. The questionnaire explored demographics; personal history; nocturnal symptoms; daytime symptoms; and Epworth sleepiness scores for hypersomnolence. Anthropometric measurements were performed as Sánchez-García et al reported [20]. Blood pressure was measured according to the American Heart Association guideline [21]. Diabetes was diagnosed using the American Diabetes Association (ADA) diagnostic criterion of fasting glucose level ≥7.0 mmol/L [22].

### Statistical analysis

All statistical analyses were performed separately for males and females. Baseline characteristics were compared between genders using Student's *t*-test for continuous variables and the chi-square test for categorical variables. To explore centralized covariant tendencies between age, BMI and WHR; and OSA severity, the distributions of these variables in terms of AHI values were initially assessed using scatter diagrams, data conversion, Pearson correlations, and stratification analysis. In these preliminary explorations, we verified the existence of linear relationships between AHI values and each risk factor (except age) via Pearson correlation analysis. To explore the existence of a possible nonlinear association between age and OSA severity, we used restricted cubic spline regression to flexibly model such association with knots placed at the 5th, 35th, 65th, and 95th percentiles. We evaluated nonlinearity using the likelihood-ratio test, comparing the fits of models including linear and cubic spline terms (selected via stepwise regression) with a model using only linear terms [23]. Restricted cubic spline analysis revealed a nonlinear correlation between age and AHI, and yielded age crossover points allowing further piecewise linear modeling. Next, we performed stepwise multiple linear regression to identify factors that significantly influenced AHI values within each age group, and calculated regression coefficients and 95% confidence intervals (CIs). The significance level (alpha) was set at 0.05. All statistical analyses were performed using Stata/SE 12.0 for Windows (StataCorp LP).

## Results

### Demographic and clinical characteristics of all subjects, grouped by gender

A total of 2,749 patients were consecutively recruited over 5 years, and 85.6%, or 2,353, met all inclusion criteria; their data are used in the following analysis. Of the 2,353 subjects, 1,975 were male and 378 female, giving a male: female ratio of 5.2∶1. Table 1 shows the demographic and clinical characteristics of all subjects. Males were significantly younger than females, and the AHI and ESS values of males significantly higher than those of females, suggesting that males had more severe OSA than did females. Males also exhibited significantly higher BMI and WHR values. Smoking and drinking were more prevalent in males. No significant difference in the prevalence of either hypertension or diabetes was evident between males and females.

**Table 1:** Baseline differences between males and females.

Variable	Male (n = 1,975)	Female (n = 378)	*p*
Age	41 (34, 50)	54 (44, 59)	<0.001
BMI (kg/m^2^)	26.97 (24.98, 29.32)	25.97 (23.43, 28.36)	<0.001
WHR	0.96 (0.93, 0.99)	0.92 (0.88, 0.95)	<0.001
Smoker, N (%)	968 (49.0)	14 (3.7)	<0.001
Drinker, N (%)	592 (29.97)	26 (6.88)	<0.001
Presence of hypertension, N (%)	489 (24.8)	90 (23.8)	0.561
Presence of diabetes, N (%)	135 (6.8)	28 (7.4)	0.659
ESS	9 (6, 14)	8 (3, 12)	<0.001
AHI	42.41 (19.46, 62.78)	23.40 (11.40, 46.55)	<0.001

Skewed data are presented as the medians (interquartile ranges), and categorical data are presented as the numbers (percentages). All ages age shown in years. Differences between males and females were examined by the Mann-Whitney U test or Chi squared test.

Abbreviations: BMI, Body mass index; WHR, waist circumference/hip circumference ratio; ESS, Epworth sleepiness score; AHI, apnea-hypopnea index.

### The distributions of variables

The continuous variables age, BMI, and WHR, all displayed abnormal distributions when plotted against OSA severity. To define more accurate correlative models linking OSA severity to various risk factors, we considered that exploration of the distribution rules for the various risk factors was essential. As we found that associations between age, BMI, and WHR; and OSA severity, differed in males and females, it was necessary to further analyze the ways in which such variables correlated with OSA severity by gender. The distribution rules were assessed using scatter diagrams, data conversion, Pearson correlation, and stratification and restricted cubic spline analyses.

**Figure 1:**
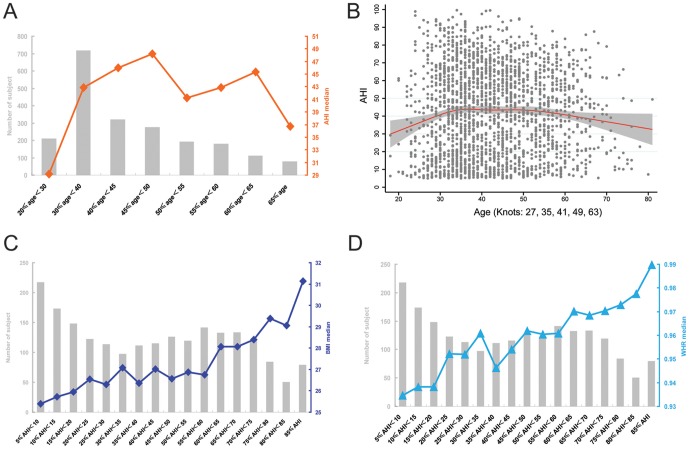
Exploration of the distribution rules for age, BMI, and WHR in male patients. The gray histogram shows the numbers of subjects in each stratification, and centralized tendencies are represented by the median values. To simplify the Figure, percentile intervals are omitted. A, OSA severity (AHI) was plotted against age stratification. B, Restricted cubic spline analysis of the dynamic change in age trend against AHI; grey dots represent raw AHI values of all patients; grey areas the 95% confidence intervals; and the red curve the overall centralized tendency of AHI against increasing age. C, BMI medians plotted against AHI severity stratifications. D, WHR medians plotted against AHI severity stratifications.

In terms of male age distribution, none of the scatter diagram, data conversion, Pearson correlation, or stratification analysis afforded clear guidance on correlations. When AHI was plotted against age stratification, OSA severity tended to increase from age ≥20 years up to age <50 years, and then decreased with instability (Figure 1A), suggests that the correlation between age and OSA severity is more complicated than current understanding [13]–[18].

To calculate the centralized covariant tendency between age and OSA severity that can represent the real distribution rule, a restricted cubic spline analysis was adopted. Restricted cubic spline analysis optimally fitted age to AHI values, and yielded crossover points allowing further piecewise linear modeling. As shown in Figure 1B, age, after adjustment for other variables (BMI, WHR, smoking, drinking, hypertension and diabetes) exhibited a multilevel linear relationship with OSA severity. The knots at ages 27, 35, 41, 49, and 63 years suggested that correlations between age and OSA severity varied among these age ranges. In other words, the association between age and OSA severity could not be fitted using an ordinal model [16]. Of the male obesity index values, both BMI and WHR increased with OSA severity, in an approximately linear manner, upon univariate plotting, regardless of the results yielded by scatter diagrams, stratification, or restricted cubic spline analyses. The Pearson correlation coefficients for BMI and WHR were 0.416 (P<0.001) and 0.229 (P<0.001), respectively (Figure 1, C and D).

**Figure 2:**
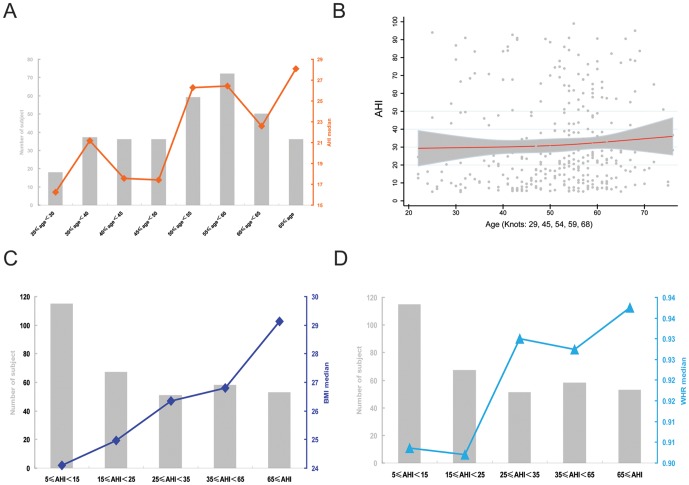
Exploration of the distribution rules of age, BMI, and WHR, in female patients. The gray histogram shows the numbers of subjects in each stratification, and centralized tendencies are represented by the median values. To simplify the Figure, percentile intervals are omitted. A, OSA severity (AHI) was plotted against age stratification. B, Restricted cubic spline analysis of the dynamic change in age trend against AHI; grey dots represent raw AHI values of all patients; grey areas the 95% confidence intervals; and the red curve the overall centralized tendency of AHI against increasing age. C, Media BMI plotted against AHI severity stratification. D, WHR medians plotted against AHI severity stratification.

In females, we sought to identify an impact of menopause on OSA severity via menopause-specific age stratification, but any correlation between age and OSA severity remained obscure. However, both age stratification and restricted cubic spline analysis revealed that the severity of OSA was higher in older than younger subjects (Figure 2, A and B). The knots for age were 29, 45, 54, 59, and 68 years upon restricted cubic spline analysis adjusted by BMI, WHR, smoking, drinking, hypertension, and diabetes (Figure 2B). Of female obesity index values, both BMI and WHR tended to increase with OSA severity, but BMI afforded a more regular (approximately linear) model fit than did WHR in this context. The Pearson correlation coefficients for BMI and WHR were 0.365 (P<0.001) and 0.294 (P<0.001), respectively (Figure 2 C and D).

### Age-group-specific associations between the severity of OSA and relevant risk factors in male and female patients

The work described above provided guidance for further statistical analysis: 1) obesity was positively associated with OSA severity in a manner that was approximately linear; and, 2) age was associated with OSA severity only at certain ages. Thus, a fitted analysis strategy that can combine these two correlation rules is needed to uncover associations between the severity of OSA and relevant risk factors. After comprehensive analysis on the centralized covariant tendency between age and OSA severity calculated by restricted cubic spline and age stratification analysis, serial multiple linear regression modeling (stratified by age) was performed to detect any correlations between age group and OSA severity. As summarized in Table 2, the male population was finally regrouped into age≤40 years and age≥41 years subgroups; and the female population into age≤44 years, 45<age≤53, and age≥54 years subgroups. The distribution characteristics of BMI, WHR, OSA severity, personal history, and disease history, which will be used as adjustment factors upon final multiple linear regression modeling, are also shown in Table 2.

**Table 2:** Baseline data used in age regrouping.

	Category	Number of subjects	BMI	WHR	Smoker	Drinker	Presence of hypertension	Presence of diabetes	AHI
***Male (N = 1,975)***									
Age groups	∼40	952 (48.2)	27.18 (25.10, 29.41)	0.95 (0.92,0.99)	451 (47.4)	273 (28.7)	207 (21.7)	30 (3.2)	41.1 (17.5, 64.7)
	41∼	1023 (51.8)	26.65 (25.10, 29.04)	0.96 (0.93,0.99)	517 (50.5)	314 (30.7)	282 (27.6)	105 (10.3)	45.1 (21.2, 63.5)
***Female (N = 378)***									
Age groups	∼44	99 (26.2)	25.24 (22.03, 28.93)	0.90 (0.86, 0.95)	10 (10.1)	12 (13.33)	19 (19.2)	8 (8.1)	19.5 (9.2, 47.7)
	45∼53	85 (22.5)	25.95 (24.03, 27.68)	0.90 (0.89, 0.94)	2 (2.4)	6 (7.79)	24 (28.2)	4 (4.7)	21.3 (12.6, 38.9)
	54∼	194 (51.3)	26.13 (23.58, 28.74)	0.92 (0.9, 0.96)	2 (1.0)	8 (4.55)	47 (24.2)	16 (8.2)	26.5 (13.9, 49.6)

To explore centralized covariant tendencies between OSA and potential risk factors, the variable distribution characteristics were assessed by serial descriptive statistical approaches. The BMI and WHR values of both male and female populations displayed approximately satisfactory linear correlation trends as OSA severity increased. Although the plot of age against OSA severity was complex, further restricted cubic spline analysis revealed four (male) and three (female) age groups showing correlations between age and OSA severity. Then, the study populations were regrouped by age group.

Abbreviations: BMI, Body mass index; WHR, waist circumference/hip circumference ratio; AHI, apnea-hypopnea index. Skewed data were presented as the median (interquartile range), and categorical data were presented as the number (percentage). All ages age shown in years.

In males, age was associated with OSA severity only in those aged <40 years, with an unstandardized partial regression coefficient of 0.39 and 95% CIs of (0.07, 0.71). In females, age was associated with OSA severity only in those aged 45–53 years, with an unstandardized partial regression coefficient of 1.07 and 95% CIs of (1.00, 1.13) (Table 3). Other risk factors were associated, in different ways, with OSA severity in various age groups. BMI was associated with OSA severity in males and females of all ages (Table 3). Interestingly, WHR was associated with OSA severity at all ages in males but was not associated with OSA severity in females of any age (Table 3). Diabetes was associated with OSA severity only in males with age≥41 years. Hypertension was not an independent risk factor for OSA severity both in male and female patients (Table 3).

**Table 3:** Factors associated with OSA severity upon multiple linear regression models stratified by age.

	Male				Female		
	∼40	41∼48	49∼62	63∼	∼44	45∼53	54∼
	N = 952	N = 446	N = 468	N = 109	N = 99	N = 85	N = 194
Age	0.39 (0.07, 0.71)	/	/	/	/	1.07 (1.00, 1.13)	/
BMI	2.08 (1.58, 2.58)	2.54 (1.89, 3.20)	1.49 (0.91, 2.08)	1.76 (0.79, 2.73)	1.08 (1.05, 1.11)	1.09 (1.04, 1.14)	1.09 (1.05, 1.12)
WHR	9.03 (5.48, 12.58)	/	7.78 (3.74, 11.82)	7.03 (1.00, 13.96)	/	/	/
Presence of hypertension	/	/	4.54 (0.26, 8.82)	/	/	/	/
Presence of diabetes	/	14.08 (6.62, 21.54)	8.87 (2.95, 14.78)	/	/	/	/

Male and female subjects were stratified into four and three groups in terms of centralized covariant tendencies between age and OSA severity determined by restricted cubic spline analysis, the risk factors assciated with OSA severity (AHI) were screened by using multiple linear regression models for each age group. The impact of each factor was expressed as an unstandardized partial regression coefficient (B) with 95% confidence interval. The B for Age or BMI indicates the unstandardized partial regression coefficient for 1 unit increased. The B for WHR indicates the unstandardized partial regression coefficient for 0.1 unit increased.

Abbreviations: BMI, Body mass index; WHR, waist circumference/hip circumference ratio; AHI, apnea-hypopnea index.

## Discussion

The pernicious nature of OSA is increasingly understood [24], and identification of OSA-related risk factors and complications of the condition are essential for effective treatment in clinical practice. Many earlier studies have shown that age, gender, BMI, WHR, and the presence of hypertension and diabetes, were all associated with OSA [11], [12], [25], [26], but few studies have derived accurate correlative models between such risk factors and OSA severity.

To determine accurate correlations, we analyzed data from 1,975 male and 378 female OSA patients ranging widely in age, BMI, WHR, personal histories, and OSA severity. Initial analysis showed that age, BMI, WHR, smoking, and drinking affected OSA severity differently in males and females, but the effect of neither hypertension nor diabetes was gender-specific. We analyzed females and males separately, using several strategies to explore centralized covariant tendencies of risk factors for OSA severity. Scatter diagram and stratification analysis showed that BMI and WHR increased, in an approximately linear manner, with OSA exacerbation in males, but, in females, although this was also true of WHR, the WHR index soared in patients with AHI values ≥25. In terms of age, although OSA severity differed in various male and female age groups, no tested approach (including logarithmic transformation) successfully extracted a generalizable centralized tendency in the context of OSA severity. However, restricted cubic spline analysis identified age knots that defined different correlations between age and OSA severity. Although such analysis showed that the effect of age decreased in older male patients and increased in older female patients, multiple linear regression modeling after adjustment for variables including BMI, WHR, and the presence of hypertension and diabetes, showed that age exacerbated OSA only in males aged ≤40 years and in females aged 45–53 years. In our male population, age was associated with OSA severity in only two age groups; OSA severity increased in those aged ≤40 years and plateaued in older males. In our female population, age was associated with OSA severity in three age groups, of which two were plateau phases (≤44 years and ≥54 years) separated by a phase of positive correlation (age 45–53 years). These findings differ from those of previous reports suggesting that age was associated with OSA either via a simple ordinally increasing model, or negatively at certain ages [13], [14], [16]–[18].

BMI was positively associated with OSA severity in an approximately linear manner upon univariate analysis, and this was verified upon multiple linear regression modeling stratified by age, after adjustment for confounding factors. Although WHR was not a risk factor for OSA severity in females, this might be attributable to uniform WHR distributions in female patients.

We focused only on OSA patients, seeking to reveal how gender, age, obesity, hypertension, and diabetes associated with OSA severity. The ages and AHI index values of our population ranged from 20–80 years and 5–100, respectively. Also, we studied 1,975 male and 378 female patients, ensuring study scope, depth, and reliability. We performed multiple linear regression modeling using raw values of continuous variables, thus avoiding any errors created during data processing. Use of such a strategy is challenging when it is sought to identify factors weakly associated with OSA, because the use of binary variables is associated with subjectivity, and might inappropriately enhance the impacts of such variables upon relative statistical modeling [27]. Other risk factors, such as dyslipidemia, were not evaluated in the present study because we consider that age, obesity and personal history are the fundamentally relevant parameters. To seek accurate and credible correlation manner between these parameters and the severity of OSA is essential for further interpretation of OSA relative consequences.

Subjects have been treating with continuous positive airway pressure were excluded from this study because: 1) in China, many outpatients with various chronic diseases are always diagnosed as late or severity stage at their first visit to hospital and the screening strategy for OSA has not been carried out. Thus, most of the patients were involved into this study before continuous positive airway pressure treatment; 2) the continuous positive airway pressure treatment will improve OSA relative physiopathologic phenotypes.

In conclusion, age associated with OSA severity with a multilevel linear relationship, further adaptive multiple linear regression modeling revealed age-group-specific risk factors associated with OSA severity in male and female patients.
